# Lipid levels and major adverse cardiovascular events in patients initiated on statins for primary prevention: an international population-based cohort study protocol

**DOI:** 10.3399/bjgpopen20X101127

**Published:** 2020-12-16

**Authors:** Joseph E Blais, Ralph Kwame Akyea, Annelize Coetzee, Amy HY Chan, Wallis CY Lau, Kenneth KC Man, Jeff Harrison, Esther W Chan, Kebede A Beyene, Ian CK Wong, Stephen Weng

**Affiliations:** 1 Centre for Safe Medication Practice and Research, Department of Pharmacology and Pharmacy, Li Ka Shing Faculty of Medicine, University of Hong Kong, Hong Kong, China; 2 Primary Care Stratified Medicine, Division of Primary Care, University of Nottingham, Nottingham, UK; 3 School of Pharmacy, Faculty of Medical and Health Sciences, University of Auckland, Auckland, New Zealand; 4 Research Department of Practice and Policy, University College London School of Pharmacy, London, UK

**Keywords:** hydroxymethylglutaryl-CoA reductase inhibitors, cholesterol, major adverse cardiovascular events, primary prevention, cohort studies

## Abstract

**Background:**

Clinical guidelines recommend specific targets for low-density lipoprotein cholesterol (LDL-C) and non-high-density lipoprotein cholesterol (non-HDL-C) for primary prevention of cardiovascular disease (CVD). Furthermore, individual variability in lipid response to statin therapy requires assessment of the association in diverse populations.

**Aim:**

To assess whether lower concentrations of LDL-C and non-HDL-C are associated with a reduced risk of major adverse cardiovascular events (MACE) in primary prevention of CVD.

**Design & setting:**

An international, new-user, cohort study will be undertaken. It will use data from three electronic health record databases from three global regions: Clinical Practice Research Datalink, UK; PREDICT-CVD, New Zealand (NZ); and the Clinical Data and Analysis Reporting System, Hong Kong (HK).

**Method:**

New statin users without a history of atherosclerotic CVD, heart failure, or chronic kidney disease, with baseline and follow-up lipid levels will be eligible for inclusion. Patients will be classified according to LDL-C (<1.4, 1.4–1.7, 1.8–2.5, and ≥2.6 mmol/l) and non-HDL-C (<2.2, 2.2–2.5, 2.6–3.3, and ≥3.4 mmol/l) concentrations 24 months after initiating statin therapy. The primary outcome of interest is MACE, defined as the first occurrence of coronary heart disease, stroke, or cardiovascular death. Secondary outcomes include all-cause mortality and the individual components of MACE. Sensitivity analyses will be conducted using lipid levels at 3 and 12 months after starting statin therapy.

**Conclusion:**

Results will inform clinicians about the benefits of achieving guideline recommended concentrations of LDL-C for primary prevention of CVD.

## How this fits in

To the authors’ knowledge, this is the first international study to assess LDL-C concentrations after initiating statin therapy for primary prevention of CVD in real-world populations. This extends the evidence obtained from randomised clinical trials. Results will inform patients and clinicians about the associated benefits of guideline recommended LDL-C and non-HDL-C targets in real-world practice.

## Introduction

CVD is the leading cause of death globally.^1^ Atherogenic lipoproteins (LDL-C and non-HDL-C) are well-established risk factors for atherosclerotic CVD.^2,3^ Recent evidence from secondary prevention trials indicates that individuals with lower LDL-C concentrations have a reduced risk of subsequent cardiovascular events.^4,5^

While lower targets for LDL-C are gaining wider acceptance for secondary prevention of CVD, there remains limited evidence for specific lipid targets for primary prevention. The recent European clinical guidelines for the treatment of dyslipidaemia emphasise specific LDL-C and non-HDL-C targets for primary prevention according to level of CVD risk.^6^ However, whether achieving a specific lipid target while taking statin therapy for primary prevention corresponds to a reduction in cardiovascular events is uncertain, as most randomised controlled trials of statin therapy have not allocated patients to specific lipid targets. It is also unknown whether there is a lipid level threshold, below which no added clinical benefit is obtained, and if a 'lower is better' approach is appropriate for primary prevention of CVD. Understanding the lowest absolute beneficial level of LDL-C is important as very-low LDL-C levels are now achievable with proprotein convertase subtilisin-kexin type nine inhibitors, and emerging treatments such as inclisiran.^4,7^ The association between on-treatment cholesterol levels and risk of cardiovascular events has been assessed, but these have mostly been in secondary prevention statin trials, and whether these findings extend to clinical practice marked by individual variability in statin response requires further investigation.^8^

Ethnic group, and consequently pharmacogenomics, influences response to statin therapy, particularly for Asians.^9^ Variability in statin response may be present for other non-Caucasian ethnic groups. Moreover, organisation of health systems,^10^ statin dosing,^11^ and individual response to treatment^12^ vary and may impact the magnitude of benefit derived from statin therapy. Given these factors, an assessment of lipid levels in diverse populations is needed to provide representative data, as most primary prevention statin trials enrolled middle-aged Caucasians.^13^ Therefore, this study has two objectives:

1. To compare the incidence of MACE in adults taking statins for primary prevention of CVD between HK, NZ, and the UK; and
2. To assess the association between LDL-C and non-HDL-C concentrations at 24 months after initiating statin therapy and MACE in adults taking statins for primary prevention of CVD, across three geographically and ethnically diverse populations.

The findings will be relevant to clinicians and organisations developing treatment guidelines worldwide.

## Method

### Study design

**Figure 1.**
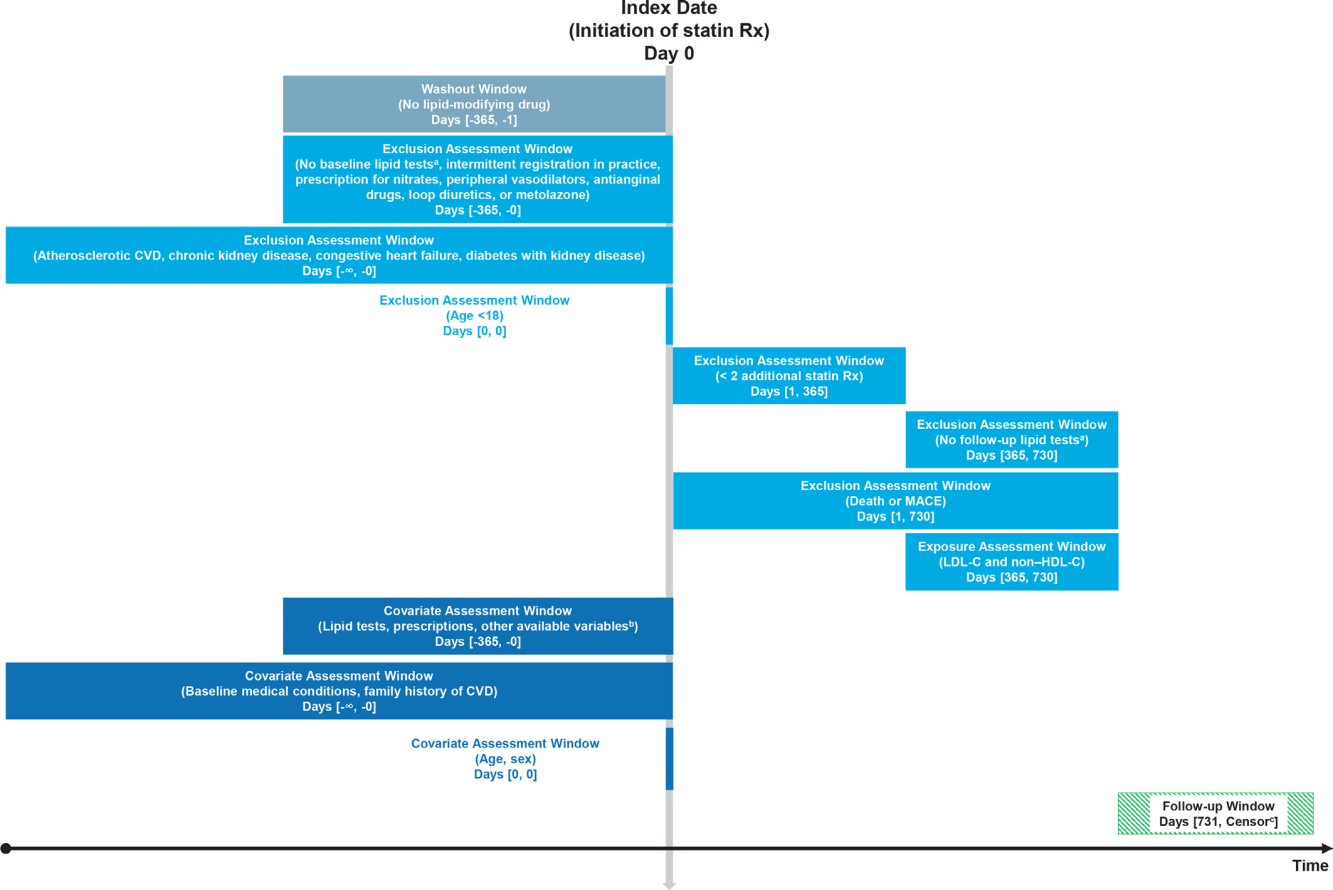
Cohort study restricted to new users of statins for primary prevention of cardiovascular disease. Figure adapted from www.repeatinitiative.org. ^25^ ^a^Defined as total cholesterol, low-density lipoprotein cholesterol, and high-density lipoprotein cholesterol. ^b^For example, body mass index, blood pressure, and deprivation index. ^c^Censored at earliest of MACE, death, disenrollment, or end of the study period. CVD = cardiovascular disease. LDL-C = low-density lipoprotein cholesterol. MACE = major adverse cardiovascular event. non-HDL-C = non–high-density lipoprotein cholesterol. Rx = prescription.

An international, multiple database study will be conducted, using a distributed network approach. Each site will follow a common protocol, to conduct an exposure-based new-user cohort study. The overall study design, study population, criteria for inclusion and exclusion, and follow-up time are shown in Figure 1. The cohort entry date (index date) is defined as the first dispensing or prescribing of a statin between 1 January 2007 and 31 December 2012. To prevent survival bias and covariate measurement bias, a new user, rather than a prevalent user, design will be used. A new statin user is defined as a patient who initiates a statin and received no lipid-modifying therapy prior to the cohort entry date. This protocol is written in accordance with the REporting of studies Conducted using Observational Routinely collected health Data for PharmacoEpidemiology statement (RECORD-PE).^14^

### Setting and data sources

Patient data will be included from electronic health record databases in three different countries. The Clinical Practice Research Datalink (CPRD) GOLD (UK) and PREDICT-CVD (NZ) are primary care databases; the Clinical Data and Analysis Reporting System (CDARS) (HK) is an integrated health system database (primary and hospital care).

CPRD contains anonymised patient data for >15 million patients and is representative of the general UK population in terms of sex, age, and ethnic group. CPRD is one of the largest quality-assured databases of longitudinal medical records from primary care in the world, with approximately 700 UK primary medical care practices (that is, covering approximately 10% of the UK population). CPRD has good ascertainment of major diagnoses, has been validated for pharmacoepidemiology studies, and has a long-established use for major research.^15^

PREDICT-CVD is a prospectively designed, open-cohort study in NZ that automatically recruits participants when primary healthcare practitioners complete standardised CVD risk assessments using PREDICT decision-support software.^16^ Approximately 95% of New Zealanders are enrolled in primary health organisations, which provide the majority of primary health care to New Zealanders nationally.^17^ About a third of the country's population is served by clinics that use PREDICT software, mainly in the Auckland and Northland regions of NZ. Participant risk factor profiles captured by the software are regularly linked to national databases documenting drug dispensing and International Classification of Diseases (ICD)-coded hospitalisations and deaths related to CVD. Data linkage from PREDICT-CVD will be performed using data of hospitalisations and emergency department (ED) visits drawn from the National Minimum Dataset (NMDS), and data from the Integrated Data Infrastructure (IDI), as well as the Mortality, National Lab Monitoring, Pharmaceutical collections (that is, record of dispensed medication based on claims data), from the Ministry of Health NZ and Testsafe databases (containing dispensing data for the northern NZ region). These health datasets contain data from different parts of the health sector, obtained from patient utilisation of health services, mandatory reporting national collections, or national population health surveys.

CDARS contains electronic health record data from the Hong Kong Hospital Authority, an integrated public healthcare system, which serves >7.4 million HK residents through 43 hospitals and institutions, 49 specialist outpatient clinics, and 73 general outpatient clinics.^18^ CDARS covers approximately 80% of all hospital admissions in HK,^19^ and has been extensively used for conducting high-quality population-based studies. Data validation has demonstrated a high-coding accuracy in CDARS.^20^

### Feasibility assessment and sample size

At the time of submission, access to the study cohorts from CDARS and CPRD has been obtained, and an application for access to PREDICT-CVD data has been submitted.

Initial assessment of the CDARS cohort of new statin users without a history of atherosclerotic CVD identified 267 243 patients. After application of all eligibility criteria, it is expected to include approximately 140 000 patients, who will have at least 3 years of follow-up after the exposure assessment window. Assuming an incidence of 10 per 1000 person years for the outcome of MACE, an estimated 4200 outcome events will be observed in the CDARS cohort. The authors' previous study using CPRD, included 165 411 patients and identified 22 798 CVD events.^12^ After pooling the three cohorts, the number of patients and events is sufficient for a two-sided significance level of 0.05, power of 0.80, and planned hazard ratio of 0.85 (see Supplementary Appendix S1).^2,21^

### Eligibility criteria

To be included in the study, each patient must meet the following eligibility criteria:

- Have at least one documented baseline lipid test for total cholesterol (TC), HDL-C, and LDL-C within a year prior to index date.
- Have at least 12 months of clinical records after registration at the practice site (CPRD) or in the database (PREDICT).
- Have no previous history of atherosclerotic CVD:
  - defined as a hospital or outpatient diagnosis of coronary heart disease (for example, myocardial infarction, ischaemic heart disease, angina, coronary artery bypass graft, percutaneous intervention), cerebrovascular disease (stroke or transient ischaemic attack), or peripheral artery disease (PAD), prior to index date; or
  - diagnosis of heart failure, chronic kidney disease, or diabetes with chronic kidney disease, due to their equivalent level of risk as people with established CVD, prior to index date; or
  - a prescribing or dispensing of at least one prescription for nitrates, other antianginal drugs, peripheral vasodilators, loop diuretics, or metolazone within a year prior to index date.
- Be aged ≥18 years on index date.
- Have at least two additional statin prescriptions 1 year following index date.
- Have at least one documented follow-up lipid test for TC, HDL-C, and LDL-C in the year prior to the start of follow-up.

Participants who die or experience MACE before the start of follow-up will be excluded. Each participant who meets the study eligibility criteria will be entered once for analysis; duplicate entries will be removed.

### Exposures

Serum concentrations of LDL-C and non-HDL-C 24 months after initiating a statin are the primary exposures of interest (Figure 1). The latest lipid test measurement for patients with multiple records for lipid tests during the exposure assessment window will be selected. If non-HDL-C has not been reported, it will be calculated as TC − HDL-C.^6^ This timeframe after statin initiation was selected as this is when a measurable benefit in reducing cardiovascular events from statin treatment occurred in randomised controlled trials.^22,23^ Furthermore, the 24-month time point ensures most follow-up cholesterol measures are captured in the electronic health records within each database.^12^

Patients will be categorised into four exposure groups according to lipid concentrations based on specific treatment targets recommended in clinical practice guidelines.^6,24^

For LDL-C:

- <1.4 mmol/l (<55 mg/dl)
- ≥1.4 to <1.8 mmol/l (≥55 to <70 mg/dl)
- ≥1.8 to <2.6 mmol/l (≥70 to <100 mg/dl)
- ≥2.6 mmol/l (≥100 mg/dl).

For non-HDL-C:

- <2.2 mmol/l (<85 mg/dl)
- ≥2.2 to <2.6 mmol/l (≥85 to <100 mg/dl)
- ≥2.6 to <3.4 mmol/l (≥100 to <130 mg/dl)
- ≥3.4 mmol/l (≥130 mg/dl).

### Outcomes and follow-up

The primary outcome of interest is MACE, defined as a diagnosis of either coronary heart disease, stroke, or cardiovascular death. Secondary outcomes include all-cause mortality and the individual components of the composite MACE outcome. Follow-up will begin after the end of the exposure assessment window (24 months after prescribing or dispensing of the first statin prescription) and will end at the earliest of outcome of interest, death, disenrollment or transfer from primary care practice, or end of the analysis period (31 December 2017).

### Statistical analysis plan

#### Data cleaning and validation

Variable definitions will be harmonised among the three sites through consensus discussion. A common analysis protocol will be created, with the aim of standardising variables in terms of measurement units and timing of measurements. For each database, the most appropriate set of operational definitions will be applied, while considering any unique aspects of the database. Then work to standardise these definitions between each database will be undertaken. Outliers for continuous exposures and covariates will be explored. Implausible values, such as negative lipid concentrations, will be removed. The distribution of continuous variables will be compared with the known distribution from a representative population survey. Multiple imputation by chained equations will be used to address missing data of continuous covariates (for example, blood pressure) and estimates pooled according to Rubin’s rules. Baseline and follow-up lipid tests will not be imputed (see eligibility criteria).

Sites will manage data access and data curation as per local policies and guidelines. Electronic health data will be stored securely and analysed locally. Anonymised patient-level data will not be shared between sites.

#### Statistical analysis

Descriptive statistics will be used to characterise the study population. Continuous variables will be summarised using mean and standard deviation or median and interquartile range, and categorical variables will be expressed as frequency and percentage. The crude incidence rates for the outcomes of interest will be estimated, reported as cases per 1000 person years with 95% confidence intervals (CIs) derived from a Poisson or negative binomial distribution as appropriate. Time-to-event analysis using Cox proportional hazards models will be used to estimate hazard ratios with 95% CIs. The proportional hazards assumption of the Cox model will be assessed. Potential confounders will be pre-specified based on expert knowledge and available literature, and adjusted for in the model. Using the common protocol, each analysis will be run locally. Estimates for each of the three sites will then be pooled by meta-analysis using a random effects model, and heterogeneity will be estimated using *I* ^2^. All statistical analyses will be performed using either Stata, SAS, or R software.

#### Subgroup analyses

To explore heterogeneity of exposure effect, subgroup analyses will be conducted by age, sex, LDL-C categories as in the FOURIER trial^4^ (that is <0.5 mmol/l, 0.5 to <1.3 mmol/l, 1.3 to <1.8 mmol/l, 1.8 to <2.6 mmol/l, and ≥2.6 mmol/l), statin adherence prior to follow-up, statin persistence, ethnic group, and prescribed statin dose intensity.

#### Sensitivity analyses

To assess the robustness of the primary analyses, the outcomes will be assessed based on earlier lipid test measurements, specifically at 3 months (exposure assessment window days [30, 90]; follow-up window [91, censor]) and 12 months (exposure assessment window [1, 365]; follow-up window [366, censor]) after initiating statin treatment.

The association of LDL-C and non-HDL-C as continuous exposures will be further assessed, rather than as categorical exposures.

#### Covariates

Each site will define covariates that are appropriate in the context of their electronic health record database. A pre-specified minimum list of potential confounders will be adjusted for. Exploratory modelling will include adjusting for additional confounders that may be available in specific databases.

The confounders to be adjusted for in each database are as follows:

- Age on index date
- Sex
- Diagnosis of hypertension
- Diagnosis of atrial fibrillation
- Diagnosis of diabetes
- Diagnosis of dyslipidaemia
- Diagnosis of obesity
- Smoking status, or prescription for nicotine replacement therapy or diagnosis of chronic obstructive pulmonary disease (COPD)
- Alcohol use (where available) or diagnosis of chronic liver disease
- Diagnosis of rheumatoid arthritis or systemic lupus erythematosus
- Diagnosis of mental illness (for example, depression, anxiety, bipolar, and/or schizophrenia)
- Prescription for any antihypertensive medication (for example, angiotensin converting enzyme inhibitors, angiotensin II receptor blockers, calcium channel blocker, beta-blocker, and/or diuretic)
- Prescription for antidiabetic drug
- Prescription for antiplatelet agents
- Prescription for oral anticoagulants.

Additional confounders that may be adjusted for subject to data source availability:

- Deprivation index
- Baseline systolic and diastolic blood pressure
- Baseline body mass index
- Family history of CVD.

### Dissemination of results

The authors of this protocol aim to publish their results in a high-impact general medical or cardiology peer-reviewed journal to ensure the findings reach a wide readership. They also plan to present the findings at relevant national and international conferences. As per the funding agreement, a final report will be submitted to the project funder (Universitas 21).

This project serves as an international platform for collaboration among researchers and supports the development and training of one doctoral (PhD) student at each of the three institutions, and will be disseminated as part of each student’s thesis. It will also set the foundation for future international collaboration and ongoing research into the safe and effective use of medicines in other therapeutic areas.

## Discussion

### Summary

This study will assess the association of LDL-C and non-HDL-C in patients taking statin therapy for primary prevention of CVD.

### Strengths and limitations

The proposed study is significant; it includes a large number of patients from three different continents, allowing for comparisons between regions and ethnic groups. The estimates obtained from pooling the results will thus be both more precise and generalisable to the millions of patients globally who use statins for primary prevention of CVD. It will not assess the effects of very low concentrations of LDL-C such as haemorrhagic stroke and new-onset diabetes mellitus. In addition, heterogeneity between healthcare systems and electronic health records is anticipated, and will be considered in the interpretation of results. For example, public health care in HK is primarily driven by hospital and specialist care, whereas primary care providers in NZ and the UK act as the first point of contact and are gatekeepers to secondary care.

### Implications for research and practice

The study will have implications for primary care clinicians, who often prescribe statins for primary prevention of CVD. Intensive monitoring of lipid response and prescribing of high-dose statins may not be practical or well adopted approaches for primary care clinicians. Not achieving guideline recommended targets may cause concern, but this concern may not be justified if very low lipid levels are not associated with a reduced risk of cardiovascular events in primary prevention of CVD. The results will generate important information about the potential benefits of achieving or not achieving current guideline recommended absolute cholesterol goals.

The evidence will also be of interest to guideline panels and policymakers, who make recommendations for the primary prevention of CVD. It will help further the understanding of the relationship of cholesterol and the future risk of CVD in three diverse international populations. By including patients from diverse ethnic groups from three regions, the results will be directly applicable to policymakers in the UK, NZ, and HK.
